# Validation of a Spanish version of the psychological inflexibility in pain scale (PIPS) and an evaluation of its relation with acceptance of pain and mindfulness in sample of persons with fibromyalgia

**DOI:** 10.1186/1477-7525-11-62

**Published:** 2013-04-18

**Authors:** Baltasar Rodero, Joao Paulo Pereira, Maria Cruz Pérez-Yus, Benigno Casanueva, Antonio Serrano-Blanco, Maria J Rodrigues da Cunha Ribeiro, Juan V Luciano, Javier Garcia-Campayo

**Affiliations:** 1 Department of Psychology. Centro Rodero, Clínica de Neurociencias, Santander, Spain; 2 Instituto Superior de Maia, Porto, Portugal; 3 Department of Psychiatry, Miguel Servet University Hospital.University of Zaragoza. Instituto Aragonés de Ciencias de la Salud (IACS), Zaragoza, Spain; 4 RheumatologyClinic. Clínica de Especialidades, Santander, Spain; 5, Parc Sanitari Sant Joan de Déu, & Fundación Sant Joan de Déu, SantBoi de Llobregat, Barcelona, Spain; 6 Red de Investigacion en Actividades Preventivas y Promocion de la Salud RD 12/0005/0006, Research Network onPreventativeActivities and HealthPromotion Instituto de Salud Carlos III, Madrid, Spain; 7 Servicio de Psiquiatría, Hospital Miguel Servet, Universidad de Zaragoza, Zaragoza, Spain

**Keywords:** Psychological inflexibility, Pain, Fibromyalgia, Acceptance, Mindfulness

## Abstract

**Background:**

Psychological flexibility has been suggested as a fundamental process in health. The Psychological Inflexibility in Pain Scale (PIPS) is one of the scales employed for assessing psychological inflexibility in pain patients. The aim of this study was to validate the Spanish version of the PIPS and secondly, to compare it to two other psychological constructs, the acceptance of pain and mindfulness scales.

**Methods:**

The PIPS was translated into Spanish by two bilingual linguistic experts, and then, back-translated into English to assess for equivalence. The final Spanish version was administered along with the Pain Visual Analogue Scale, Fibromyalgia Impact Questionnaire, Hospital Anxiety Depression Scale, Pain Catastrophizing Scale, Chronic Pain Acceptance Questionnaire and the Mindful Attention Awareness Scale, to 250 Spanish patients with fibromyalgia. Face validity, construct validity, reliability (internal consistency and test-retest) and convergent validity were tested. Also a multiple regression analysis was carried out.The usual guidelines have been followed for cross-cultural adaptations.

**Results:**

Data were very similar to the ones obtained in the original PIPS version. The construct validity confirmed the original two-components solution which explained 61.6% of the variance. The Spanish PIPS had good test-retest reliability (intraclass correlation coefficient 0.97) and internal consistency reliability (Cronbach’s alpha: 0.90). The Spanish PIPS’ score correlated significantly with worse global functioning (*r* = 0.55), anxiety (*r* = 0.54), depression (*r* = 0.66), pain catastrophizing (*r* = 0.62), pain acceptance (*r* = −0.72) and mindfulness (*r* = −0.47), as well as correlating modestly with pain intensity (*r* = 0.12). The multiple regression analyses showed that psychological inflexibility, acceptance and mindfulness are not overlapped.

**Conclusions:**

The Spanish PIPS scale appears to be a valid and reliable instrument for the evaluation of psychological inflexibility among a sample of fibromyalgia patients. These results ensure the use of this scale in research as well as in clinical practice. Psychological inflexibility measures processes different from other related components such as acceptance and mindfulness.

## Background

Over the last few decades, Cognitive Behavioral Therapy (CBT) has become the most commonly standard psychological treatment for chronic pain patients who have to deal with psychological distress and disabilities [1]. Although there is good evidence supporting the benefits of CBT techniques [2] the process by which it is effective is still rather unclear [3].

In recent years, there has been growing interest in Contextual Therapies, and specifically in the field of chronic pain, interest has grown in the Acceptance and Commitment Therapy (ACT) [4]. In this type of treatment, patients are asked to behave according to their personal values, emphasizing the acceptance of their private events (thoughts, emotions, bodily sensations) with openness and receptiveness as mere observers [5]. Conversely, when people are unwilling to remain in contact with their negative psychological experiences (e.g. pain, fear, and anxiety) it is seen as an important determinant of emotional turmoil and ineffective living. Mainly, two psychological processes arise: experiential avoidance and cognitive fusion [6]. Experiential avoidance is a process whereby an individual deliberately attempts to change the form or frequency of private experiences (e.g., bodily sensations, emotions, thoughts, memories, and behavioral predispositions), and the contexts in which they occur, regardless of the resultant social, emotional, cognitive and behavioral consequences [6,7]. Cognitive fusion, which supports experiential avoidance, occurs when negative thoughts and emotions have an excessive, or inappropriate, impact on behavior/valued action [6]. *Psychological inflexibility* appears when these two processes dominate an individual’s experience.

The expressed goal of ACT is to improve functioning by increasing psychological flexibility, defined as the ability to act effectively in accordance with personal values in the presence of negative private experiences such as pain or distress [4]. Psychological flexibility includes six related constructs: acceptance, contact with the present moment, values, committed action, self-as-context, and cognitive defusion [6]. Up to now, within chronic pain settings, initial evaluations of the ACT model have focused almost exclusively on the acceptance component, and the results have indicated its usefulness in improving functioning in people with chronic pain [8-11], as well as in people with pain and on stress-related sick leave [12].

Psychological flexibility is the psychological construct that captures the overarching ACT model in its most current form [13,14]. Accordingly, the assessment of individual differences in psychological flexibility is a central focus of ACT research. The Acceptance and Action Questionnaire (AAQ) was the first instrument developed for this purpose [15], but Wicksell and colleagues [16] extended this approach to persons with chronic pain. Factor analysis of the main tool for measuring Psychological Inflexibility – the Psychological Inflexibility in Pain Scale (PIPS) – revealed 38 initial items and four components: avoidance, acceptance, cognitive fusion and values orientation. Based on the evaluation of the psychometric properties of these four subscales, however, Wicksell reduced the PIPS to only 12 items and 2 subscales (avoidance and cognitive fusion) [17]. Analyses supported the reliability and validity of a two factor solution and the questionnaire demonstrated good internal consistencies, as measured by Cronbach´s alpha (.87 for the total scale, .89 and .66 for the two subscales respectively).

Although the results generally show that Psychological Flexibility is associated with reports of less pain intensity and interference, less anxiety and depression and better physical and mental functioning [16,17], the PIPS has also been utilized as an evaluative instrument. Indeed, reducing psychological inflexibility was recently found to mediate improvements in pain disability, fibromyalgia impact, the mental dimension of health related quality of life, self-efficacy, depression and anxiety, in a fibromyalgia sample [18]. However, the PIPS may also be used to identify different clusters of pain patients. The information provided could subsequently guide the clinician in tailoring intervention to address the patient´s difficulties.

These results implied the potential of improved outcomes of focusing on psychological inflexibility for chronic pain management. The aim of this paper is to validate the Spanish version of the PIPS in patients suffering from fibromyalgia. For this purpose, face validity, construct validity, reliability (internal consistency and test-retest) and convergent validity were tested. This validation will enable us to research the construct in Spanish populations as well as expand our limited knowledge concerning psychological inflexibility.

## Materials and methods

### Participants

We recruited patients from primary care settings, who were assessed at the Somatoform Disorders Fibromyalgia Unit at Miguel Servet University Hospital, Zaragoza, Spain, during 2011. The inclusion criteria were as follows: Patient ages ranged from 18 to 65 years and patients agreed to participate and fulfill the American College of Rheumatology criteria for primary fibromyalgia [19], according to a diagnosis made by a Spanish National Health Service rheumatologist. The sample size was calculated according to the recommended 10:1 ratio for number of subjects to number of test items [20]. The exclusion criteria were any medical or psychiatric disorders that would impede the patient from answering the questionnaire correctly, a predominance of chronic fatigue syndrome symptoms, and poor knowledge of the Spanish language. The study’s questionnaires and protocol were approved by the Ethical Committee of the regional health authority, and the patients signed a consent form attesting to their willingness to participate.

After consenting to the study, the recruited patients were administered a battery of questionnaires, including a pain form for demographic and pain-related variables, including the translated Spanish version of the PIPS to be validated, a Pain Visual Analogue Scale (PVAS) for pain intensity, the validated Spanish versions of the Fibromyalgia Impact Questionnaire (FIQ), the Hospital Anxiety and Depression Scale (HADS), the Pain Catastrophizing Scale (PCS), the Chronic Pain Acceptance Questionnaire (CPAQ), and the Mindful Attention Awareness Scale (MAAS).

### Measures

#### Background variables

Background information from participants included age, gender, level of education (primary school, secondary school, university), and duration of pain.

#### Psychological inflexibility in pain scale (PIPS)

The PIPS is a 12-item scale designed to measure psychological inflexibility in pain patients. Two studies have supported a 2-factor solution (avoidance and cognitive fusion related to pain) with satisfactory statistical properties [16,17]. The items consisted of different statements that were considered to be related to chronic pain, psychological inflexibility, suffering and disability (coherent with the ACT theory). All of the items were rated on a 7-point Likert-type scale that ranged from “1=never true” to “7=always true”, with higher scores indicating more psychological inflexibility.

#### Fibromyalgia impact questionnaire (FIQ)

The FIQ is a 10-item self-report questionnaire that measures the health status and global functioning in patients with fibromyalgia [21]. The first item focuses on patients’ ability to perform physical activities. The following two items require the patients to indicate the number of days in the past week they felt good and how many days of work they had missed. Finally, the last seven questions (ability to work, pain, fatigue, morning tiredness, stiffness, anxiety, depression) are measured using a visual analogue scale. A higher score indicates a worse health status and functioning. This instrument also has a translated and validated Spanish version [22].

#### Hospital anxiety and depression scale (HADS)

The HADS [23] is a self-report scale that screens for the presence of depression and anxiety in patients with "medical conditions”. The scale comprises 14 items that are rated on a 4-point Likert-type scale, and it is appropriate for use in community and hospital settings. Two subscales assessed depression and anxiety independently (HADS-Dep and HADS-Anx, respectively). The HADS was previously validated in a Spanish population [24]. The HADS was selected for use in the present study because it is considered to be one of the best questionnaires for assessing depression and anxiety in patients with pain disorders.

#### Pain visual analogue scale (PVAS)

The PVAS was designed to allow for a subjective assessment of pain. It consists of a 10 cm long straight line whose extremities represent the limits of pain intensity (“no pain” to “maximum pain ever experienced”). The patients estimated the pain intensity experienced on the same day between 0 and 100. Previous studies have demonstrated PVAS to have adequate psychometric properties [25].

#### Pain catastrophizing scale (PCS)

The PCS is a 13-item self-report questionnaire that comprises three dimensions: (a) rumination, (b) magnification and (c) helplessness. Each item is scored from 0 (not at all) to 4 (always), and scores range from 0 to 52. Its validity and reliability have been previously reported [26]. The Spanish version of the PCS has been validated by the current study's authors and shows psychometric properties similar to those of the original questionnaire [27].

#### The mindful attention awareness scale (MAAS)

The MAAS [28] is a 15-item measure of mindfulness. The item content was designed to reflect the opposite of the construct of mindfulness, or “mindlessness,” and thus endorsing the item content at a lower frequency is taken to mean a higher level of mindfulness. Each item is rated on a scale from 1 (almost always) to 6 (almost never) in relation to respondent’s “everyday experience,” and there is no specified time frame for these ratings. The item ratings are averaged to form the total score. The scale has been recently validated in Spanish showing appropriate psychometric parameters [29].

#### The chronic pain acceptance questionnaire (CPAQ)

The CPAQ [30] is a 20-item measure of acceptance of pain. It includes two components: Activity Engagement and Pain Willingness, thus reflecting acceptance as including behavioral qualities of continuing activities in the presence of pain and the absence of pain avoidance responses. Patients rate each item on a scale of 0 (never true) to 6 (always true). Previous studies indicated adequate reliability and validity for the scale. The Spanish version of the CPAQ has been validated by our team and achieves adequate reliability [31].

### Validation process

Permission to translate and validate the PIPS was obtained from the original authors [17]. Although the PIPS scale was originally conceived in Swedish we decided to validate the English version as being the most frequently found among scientific literature. Two researchers, who were aware of the objectives of the questionnaire, made the initial translation into Spanish. Each researcher translated the questionnaire separately. Subsequently, two bilingual linguistic experts, who had no specific knowledge regarding the instrument, carried out back-translations. Finally the two English versions were determined to be equivalent by a native English teacher. Any differences between the translations were resolved by mutual agreement. Both translators and authors were present during the agreement. The authors are familiar with both reading and writing technical English and are very familiar with the psychological construct being assessed with the questionnaire. The usual guidelines have been followed for cross-cultural adaptations [32]. This paper is part of broader research on psychological constructs in fibromyalgia and their validation in Spanish [33-35]. The final Spanish version is shown in Table 1.

**Table 1:** Spanish PIPS

	**1**	**2**	**3**	**4**	**5**	**6**	**7**
	**Nuncacierta**	**Muyraramentecierta**	**Raramentecierta**	**A vecescierta**	**A menudocierta**	**Casisiemprecierta**	**Siemprecierta**
1. Cuando tengo dolor cancelo las actividades que tengo planeadas	1	2	3	4	5	6	7
2. Digo cosas como: “No tengo fuerzas”, “no me siento lo bastante bien”, “no tengo tiempo”, “no me atrevo”, “tengo demasiado dolor”, “me siento bastante mal”, o “no me apetece”	1	2	3	4	5	6	7
3. Necesito saber que está mal para poder seguir adelante	1	2	3	4	5	6	7
4. Ya no hago planes de futuro debido al dolor	1	2	3	4	5	6	7
5. Evito hacer cosas cuando creo que existe un riesgo de dañarme o de que las cosas empeoren	1	2	3	4	5	6	7
6. Es importante comprender cuál es la causa de mi dolor	1	2	3	4	5	6	7
7. Para evitar tener dolores, dejo de hacer cosas que son importantes para mi	1	2	3	4	5	6	7
8. Debido al dolor pospongo cosas	1	2	3	4	5	6	7
9. Haría casi cualquier cosa para eliminar el dolor	1	2	3	4	5	6	7
10. Mi vida la controla el dolor y no yo	1	2	3	4	5	6	7
11. Debido al dolor, evito programarme actividades	1	2	3	4	5	6	7
12. Es importante que aprenda a controlar mi dolor	1	2	3	4	5	6	7

Instrucciones: A continuación, encontrará una lista de afirmaciones. Puntúa cada una de ellas haciendo un círculo en el número que mejor defina la frecuencia con la que dicha información es cierta para usted.

Evitación = sume los apartados 1, 2, 4, 5, 7, 8, 10, 11.

Fusión Cognitiva = sume los apartados 3, 6, 9, 12.

Assessments took place at two different points over a 1–2 week interval. There is no evidence available to aid in the selection of the time interval between questionnaire administrations for a study of test-retest reliability for health status instruments [36]. This time interval was selected because it was believed that the interval was too short for clinical change to occur. The subsample for the second assessment was randomly selected.

### Statistical analysis

To determine the suitability of the data for principal components analysis, the Kaiser-Meyer-Olkin Measure of Sampling Adequacy (KMO) [37] and Bartlett’s Test of Sphericity [38] were calculated. A principal components analysis was then performed to determine whether the 12 items on the scale could be combined into separate components. Varimax rotation was performed to minimize the complexity of loadings for each component. Criterion validity of the PIPS-Spanish was examined by calculating the correlations between the total PIPS-Spanish score with the PVAS, FIQ, HADS, PCS, CPAQ, and the MAAS, using Pearson’s *r* correlation coefficient. The internal consistency of the questionnaire was determined using Cronbach’s alpha. Item-total correlations were also inspected to determine the item internal consistency. Test-retest reliability, evaluated with the intraclass correlation coefficient, was assessed for the 1- to 2-week follow-up interval, during which time the patients did not change their baseline treatment.

To confirm that psychological inflexibility, acceptance and mindfulness are different constructs, and to provide data regarding their relative contributions to functioning and health variables, a multiple regression analysis was performed. Hence five regression equations were calculated. In each of these equations, the potential predictors were tested hierarchically. Firstly, patient age, sex, education, and duration of pain were tested and retained in the equations when significant (*p* < .05 to enter, *p* > .10 to remove). Secondly, pain intensity was entered to control its contribution to the prediction of each criterion variable (except in the case where pain was the criterion variable). Thirdly, the three process scores for the acceptance of pain, mindfulness and psychological inflexibility were entered together in a single block to examine their contribution. Finally, the relative role of the three separate processes was gleaned from standardized regression coefficients and from the squared semi-partial correlation coefficients. Lastly, we also attempt to address one of the hypotheses raised by Wicksell regarding the identification of discrete subgroups using the two PIPS subscales [17]. The identification of different clusters of pain patients may benefit from individually tailored interventions with different emphasis. For this purpose, a series of hierarchical and *k*-means cluster analyses were conducted. A number of cluster solutions were considered, ranging from two to four subgroups. Firstly, demographic differences among the groups were tested via chi-square or Analyses of Variance (ANOVA’s). In order to explore the potential clinical utility of the clusters, a second series of one-way ANOVA’s was conducted using cluster membership as the independent variable and the scores on the seven measures of functioning as the dependent measures. These comparisons used a Bonferroni-corrected alpha (alpha = .05/number of tests or .05/7 = .007). All statistical analyses were performed with SPSS software, Release 15 (SPSS Inc. Chicago, Illinois).

## Results

### Characteristics of the sample

Nine patients were ruled out from the study because of a predominance of their chronic fatigue syndrome symptoms. Of the 253 potential subjects, three (1.1%) declined to participate. None of the participants were ruled out because of the exclusion criteria. The final study sample consisted of 250 patients, 240 (95.6%) women and 10 (4.0%) men, aged 31–70 (mean 52.4, SD: 8.5 years), all self-described as White European. The ratio of women/men is higher in the sample reflecting a similar ratio in the prevalence of fibromyalgia in either gender. Most of the patients were married (73.3%; single 9.2%, divorced 12.7%, widowed 4.4%). Regarding education, nearly half of the sample had attended primary school education (46.2%; secondary 37.5; other 12.7%). On average, the patients who participated in the study had suffered from fibromyalgia for 18.3 years (range 1–57; SD: 11.2 years). A significant proportion of the subjects were retired, not working, or working part-time due to their pain (full-time work 25.1%, retired 13.5%, homemaker 12.7%, unemployed 15.1%, permanent disability pension 21.1%; other 12.0%).

The mean PIPS total score was 57.1 (SD 18.2, range 12–84). This amounted to a mean item rating of 4.7, which corresponds with a medium-high range of the 1–7 scale and the rating category between “Sometimes true” and “Often true” for the average psychological inflexibility item. There was not a significant association between the PIPS’ total score and most demographic characteristics including gender, age, marital status, duration of pain, education level or work status.

### Face validity

For assessing face validity, a sample of patients (N = 150) randomly recruited from the Spanish Association of Fibromyalgia was asked whether they thought that the test could adequately measure their psychological inflexibility. A total of 94% (141 out of 150) of them agreed.

### Principal components analysis

The KMO was found to be 0.91, which exceeds the recommended minimum value of 0.60. Bartlett’s Test of Sphericity was highly significant (χ^2^ = 1594, *p* < 0.001), supporting the suitability of the data for a principal components analysis. A principal components analysis (PCA) with varimax rotation yielded a two-component solution with eigenvalues greater than 1. The first component, labeled avoidance, accounted for 50.5% of the total variance. The second component, labeled cognitive fusion, accounted for 11.1% of the total variance. The loadings of the pattern matrix are presented in Table 2. The coefficient alpha for the total PIPS was 0.90.

**Table 2:** Principal component analysis of the PIPS-Spanish

**PIPS Item**	**Factor 1**	**Factor 2**	**M**	**SD**	**Item-total r**	**Cronbach’s α if item deleted**
11. I avoid scheduling activities because of my pain.	.82	.20	4.24	2.37	.76	.89
10. It’s not me that controls my life, it’s my pain.	.80	.14	4.05	2.42	.72	.89
4. Because of my pain, I no longer plan for the future.	.78	.25	4.46	2.41	.75	.89
7. I don’t do things that are important to me to avoid feeling my pain.	.77	.29	4.13	2.26	.76	.89
1. I cancel planned activities when I am in pain.	.77	.15	4.84	2.04	.69	.89
8. I postpone things on account of my pain.	.76	.32	4.71	2.05	.76	.89
5. I avoid doing things when there is a risk it will hurt or make things worse.	.71	.24	4.85	2.10	.68	.89
2. I say things like ”I don’t have any energy”, ”I am not well enough”, ”I don’t have time”, ”I don’t dare”, ”I have too much pain”, ”I feel too bad”, or ”I don’t feel like i	.61	.31	4.81	1.84	.62	.89
6. It is important to understand what causes my pain.	.12	.82	5.11	2.28	.41	.90
3. I need to understand what is wrong in order to move on.	.21	.78	4.44	2.47	.47	.90
9. I would do almost anything to get rid of my pain.	.22	.70	5.35	1.95	.54	.90
12. It is important that I learn to control my pain.	.18	.61	6.11	1.65	.38	.90

Note: Values in bold are factors loadings greater than or equal to .50.

### Internal consistency

Cronbach’s α calculation for the 12 items in the PIPS-Spanish was 0.90 (95% CI: 0.88- 0.92), indicating a high degree of internal consistency. Cronbach’s α for Factor 1 and Factor 2 were 0.92 and 0.61 respectively. All corrected item-total correlations were above 0.30 (median = 0.62, range 0.38 – 0.77), which is the cut-off criterion established by De Vellis [39].

### Test-retest reliability

The response to the PIPS-Spanish provided by a random subsample of 141 patients with fibromyalgia (gender female: 135, 95.7%; age: mean 51.7 years, SD: 8.8 years; duration of the disorder: mean 18.5 years SD: 11.3 years; and 36, 25.5% granted an invalidity pension) showed a satisfactory temporal stability of the scale over a 1–2 week interval, during which the patients did not change their baseline treatment. The test-retest correlation assessed with the intraclass correlation coefficient was 0.97 (Factor 1 = 0.96 and Factor 2 = 0.95).

### Intercorrelations between PIPS-Spanish, pain, global functioning, depression, anxiety, catastrophizing, acceptance and mindfulness

The Pearson correlation was used to assess the relationship between the PIPS-Spanish and other psychometric instruments, and the results are summarized in Table 3. Both the PIPS total score and the two subscales were significantly correlated with practically all of the other psychometric instruments, including the pain intensity, global functioning, depression, anxiety, catastrophizing, acceptance and mindfulness. Notably, however, regarding the pain intensity, the correlation showed by the PIPS-total was modest (*r* = 0.12, *p* = 0.043), whereas the subscale for cognitive fusion was non-correlative.

**Table 3:** C**orrelation between Spanish version of PIPS scores (total and subscales) and other Spanish instruments**

**Instruments**	**PIPS**	**PIPS subscales**	
	**Total score**	**Avoidance**	**Cognitive fusion**
PVAS	.12^*^	.15^*^	.024
FIQ	.55^**^	.56^**^	.36^**^
HADS-anx	.54^**^	.53^**^	.42^**^
HADS-dep	.66^**^	.66^**^	.47^**^
PCS	.62^**^	.63^**^	.42^**^
CPAQ	-.72^**^	-.73^**^	-.50^**^
MAAS	-.47^**^	-.45^**^	-.37^**^

Note: PVAS, Pain Visual Analogue Scale; FIQ, Fibromyalgia Impact Questionnaire; HADS-anx, Anxiety; HADS-dep, Depression; PCS, Pain Catastrophizing Scale; CPAQ, Chronic Pain Acceptance Questionnaire ; MAAS, Mindful Attention Awareness Scale.

^*^ Significant: P < 0.05.

^**^ Significant: P < 0.01.

### Multiple regression analyses

The correlations of the total PIPS with acceptance (*r* = −0.72, *p* < 0.01) and with mindfulness (*r* = −0.47, *p* < 0.01) were particularly high, as one could expect since they are psychological constructs derived from acceptance-based interventions. This raised concerns that psychological inflexibility might be largely redundant with these two constructs. The regression results are shown in Table 4.

**Table 4:** Multiple regression analyses of acceptance, mindfulness, and psychological inflexibility with measures of functioning and well-being

**Block**	**Predictor**	**Beta (final)**	**ΔR**^**2**^	**sr**^**2**^	**Adj total R**^**2**^
PVAS					
1.	Psy-Inflexibility	.12^*^	.01^*^	.016	.01^*^
FIQ					
1.	Age	.02	.03^**^	.00072	
2.	Pain intensity	.23^***^	.10^***^	.054	
3.	Acceptance	-.35^***^	.35^***^	-.057	
	Mindfulness	-.23^***^		-.041	
	Psy-Inflexibility	.14^*^		.0092	.48^***^
HADS-Anx					
1.	Age	.062	.05^***^	-.0034	
2.	Pain intensity	.15^***^	.06^***^	.0023	
3.	Acceptance	-.18^**^	.37^***^	-.0015	
	Mindfulness	-.39^***^		-.11	
	Psy-Inflexibility	.19^**^		.016	.47^***^
HADS-Dep					
1.	Age	.042	.02^*^	.0016	
2.	Pain intensity	.11^**^	.04^**^	.011	
3.	Acceptance	-.38^***^	.52^***^	-.067	
	Mindfulness	-.28^***^		-.058	
	Psy-Inflexibility	.24^***^		.025	.58^***^
PCS					
1.	Age	.045	.05^***^	.0016	
	Duration of pain	.12^**^	.02^*^	.012	
2.	Pain intensity	.09^*^	.03^**^	.0082	
3.	Acceptance	-.42^***^	.46^***^	-.084	
	Mindfulness	-.17^***^		-.022	
	Psy-Inflexibility	.21^**^		.019	.57^***^

*Note:* Age, sex, years of education, and duration of pain were tested as predictors in the first entry block in each equation and retained when significant (*p*<.05 to enter, *p*>.10 to remove). A 0–100 rating of pain intensity was entered in the next block. The three primary psychological variables were entered simultaneously, with a standard entry procedure, in the final block in each equation. The squared semi-partial correlation coefficient in column five (*sr*^2^) reflects the unique contribution (proportion of variance) from each predictor to the dependent variable. PVAS, Pain Visual Analogue Scale; FIQ, Fibromyalgia Impact Questionnaire; HADS-anx, Anxiety; HADS-dep, Depression; PCS, Pain Catastrophizing Scale; CPAQ, Chronic Pain Acceptance Questionnaire ; MAAS, Mindful Attention Awareness Scale.

^*^p< .05. ^**^p< .01.^***^p< .001.

### Cluster analysis

Upon review of these analyses, it was apparent that there were three distinct clusters of patients. Indicatively, two of the clusters had either high or low scores on both of the subscales (n’s = 93 and 77, respectively). The third (*n* = 81) had scores that were discordant in that they demonstrated a tendency that was slightly lower on the Avoidance subscale and high on the Cognitive Fusion subscale. The PIPS scores for each cluster differed significantly from one another (see Table 5), although these differences should be interpreted with a degree of caution given that the cluster analytic procedure was designed to maximize them.

**Table 5:** Mean (SD) PIPS scores by fibromyalgia clusters

	**Entire sample *(N*=250)**	**Clusters**		
		**High A and CF ( *n *=92)**	**Medium A and high CF ( *n *=81)**	**Low A and CF ( *n *=77)**
Avoidance (A)^*^	36.29 (14.1)	50.88 (3.9)	35.62 (4.1)	18.27 (5.2)
Cognitive Fusion (CF)^*^	20.81(5.7)	24.29 (4.3)	22.40 (3.9)	16.02 (5.5)
Total Score	57.10 (18.2)	75.17 (6.5)	58.02 (6.3)	34.29 (8.5)

^*^Pairwise comparisons of the PIPS total and subscale scores were all significantly different among the clusters. These differences should only be used descriptively as the cluster analytic procedure was designed to maximize them.

No significant demographic differences were found between the groups. Table 6 displays the results of these analyses, as well as descriptive information. The following pattern of findings emerged. Firstly, the high scoring PIPS cluster significantly differed from the low scoring cluster in six out of seven measures with the high scoring group reporting more pain, fibromyalgia impact, depression, anxiety, catastrophizing as well as less pain acceptance and mindfulness. The scores of the third cluster generally fell in between the scores of the other two and were significantly different from both clusters in six of the analyses. Pain intensity was the only measure that did not show any significant difference among the groups.

**Table 6:** Mean (SD) scores on the measures of functioning and well being by clusters

	**Entire sample *(N*=250)**	**Clusters**		
		**High A and CF**	**Medium A and high CF**	**Low A and CF**
PVAS	52.5 (16.9)	55.5 (16.4)	51.3 (17.1)	50.0 (16.7)
FIQ	58.1 (15.0)	66.6 (12.2)^*^	58.1 (12.8)^*^	47.3 (13.4)^*^
HADS-anx	10.8 (5.0)	13.4 (4.1)^*^	10.7 (4.6)^*^	7.5 (4.3)^*^
HADS-dep	7.7 (4.7)	10.8 (4.1)^*^	7.7 (3.7)^*^	3.8 (3.1)^*^
PCS	24.3 (13.6)	33.2 (12.1)^*^	24.0 (11.1)^*^	13.8 (9.4)^*^
CPAQ	47.6 (23.4)	30.9 (14.5)^*^	45.5 (16.7)^*^	69.7 (20.1)^*^
MAAS	3.7 (1.3)	3.3 (1.1)^*^	3.5 (1.0)^*^	4.5 (0.96)^*^

Note: PVAS, Pain Visual Analogue Scale; FIQ, Fibromyalgia Impact Questionnaire; HADS-anx, Anxiety; HADS-dep, Depression; PCS, Pain Catastrophizing Scale; CPAQ, Chronic Pain Acceptance Questionnaire ; MAAS, Mindful Attention Awareness Scale.

^*^Significant differences at *p*< .007.

## Discussion

The main purpose of the present research was to validate the Spanish version of the Psychological Inflexibility Pain Scale (PIPS-Spanish) in patients with fibromyalgia and, in addition, to examine the impact of the PIPS in fibromyalgia compared with other psychological constructs, and to identify potential subgroups using the PIPS.

The results mainly suggest that the PIPS might provide an additional useful tool to assess psychological risk for problematic outcomes in fibromyalgia. The PIPS-Spanish showed high internal consistency and high test-retest reliability, as well as significant correlations with associated constructs such us pain severity, global functioning, anxiety, depression, catastrophizing, acceptance and mindfulness. Furthermore, the Scree plot indicated a two-factor construct of the translated questionnaire similar to its original English version. Both factors had eigenvalues greater than one. Principal components with varimax rotation revealed a satisfactory percentage of Total Variance explained (61.6%) by the two factors. Looking at the component matrix of the two-factor construct, individual items could be allocated to the same subscales as they were in the English version. Therefore, construct validity of the translated PIPS can be supported.

Results showed that patients with lower psychological inflexibility were associated with better global functioning and well-being. Interestingly, the correlation between the PIPS and its subscales in terms of pain intensity was modest or non-existent. This might be due to the fact that ACT scales are mainly designed to measure functioning and wellbeing. The current results are consistent with previous studies from other contextual constructs such as acceptance or mindfulness and their relatively low correlations with pain intensity [40-42]. In other words, these results from contextual processes would imply that functioning and well-being do not depend directly upon the pain intensity.

The correlations among these three ACT constructs were strong and the results from multiple regression analyses illustrate their relevant, combined contribution to explained variance in global functioning and well-being. More importantly, the findings also confirmed that there was no overlap among these processes. Indeed, each one of them made significant contributions to the outcomes. This study was not designed to identify which is the most important construct, but in comparison with the other two processes, acceptance of pain seems to achieve slightly higher correlations with the measures of functioning and health.

Specifically it is somewhat surprising that CPAQ and PIPS are not overlapped. The reason seems to be that although items in CPAQ are clearly related to avoidance and cognitive fusion, PIPS constitutes an attempt to refine the assessment of these specific subcomponents of psychological inflexibility and to produce a measure with clearly discernible factors of avoidance and cognitive fusion. Indeed, data from the present study support the fact that PIPS provides a measure of processes previously not quantified in pain patients.

Cluster analyses rendered additional support for the two-factor model and indicated three discrete patient groupings. The first two were as expected and contained individuals who were either high or low on both subscales. In subsequent comparisons, these two groups differed statistically in six out of the seven measures of functioning and well-being. In four out of six measures, with the exception of fibromyalgia impact and mindfulness, the high PIPS group reported difficulties with emotional disturbances or functioning that were approximately twice those reported by the low group. These results highlight the need for adequate treatments for those with fibromyalgia who are particularly unable to be active for reasons of pain and struggle unsuccessfully for control over pain.

Based on our clinical experience, the presence of the third cluster was somewhat expected. This cluster demonstrates that individuals who do not present for treatment report an exaggerated avoiding pattern, but at the same time they identify a strong need for pain control. In fact, these individuals may report that they were functioning well to a degree; however, in the present data they also report significant disturbances in wellbeing and functioning. It is possible that this particular group would benefit from treatment to enhance techniques that allow the individuals to disentangle from thoughts. Perhaps these same individuals would also benefit from treatment that paid somewhat less attention to methods for increasing physical activity specifically, although future investigation will be necessary to explore the accuracy of this hypothesis.

Notably, high scores on the avoidance subscale did not co-occur with low scores on the Cognitive Fusion subscale. This is coherent with what ACT proposes that the individual is trapped by self-barriers limiting their lives. People who experience cognitive fusion tend to misinterpret “thoughts” as facts, therefore serving as excuses for their behaviors. Patterns of discordance between the two PIPS subscale scores deserve further study. Previous studies have shown the relevance of targeting “Cognitive Fusion” [42-44]. The present findings may indicate that tailoring this component adds unique benefits in treatment reinforcing previous data. Longitudinal designs will be useful to empirically test this possibility.

The current study has a number of limitations that call for a cautious interpretation of some of the results. Firstly, our correlation methods cannot unambiguously determine whether psychological inflexibility leads to decreased levels of functioning and wellbeing or vice versa. Secondly, the main target of this study was to validate the scale so it might be that the total sample for the cluster was limited. Lastly, the processes examined are technically complex to measure and, in many ways, the instruments being used are relatively recent developments.

Indeed, currently, there is some ongoing critical discussion within the ACT research field in regards to the processes underlying psychological flexibility. For instance, the PIPS as well as another questionnaire, the Brief Pain Coping Inventory [45,46], consider psychological flexibility as the sum of different components, for that reason they were designed to find various factors. However, recent research using a different scale to assess psychological flexibility as the AAQ-II, rejects a two-factor solution and considers the construct as an unidimensional measure [13]. Certainly, further experience may lead us to refine these instruments, and different or better instruments may reveal a different pattern of results.

## Conclusions

The Spanish version of the PIPS scale has been shown to be a valid and reliable instrument for measuring psychological inflexibility in patients with fibromyalgia. Although psychological inflexibility is considered to be one of the key treatment processes influencing change in primary outcome variables e.g. pain disability, there have been hardly any studies to enhance our knowledge of this concept. This study will make it easier to assess psychological inflexibility in Spanish populations. Secondly, psychological inflexibility measures different processes than other ACT components such as acceptance or mindfulness. Finally, the PIPS might be used to identify different clusters of fibromyalgia patients that may benefit from individually tailored interventions with different areas of emphasis.

## Competing interests

The authors declare that they have no competing interests.

## Authors’ contributions

JGC, BR, JPP and MJRCR are the principal researchers and developed the original idea for the study. ASB, BC and MCPY participated in the design and planning of the intervention that is evaluated here. JVL developed the statistical methods. All authors have read and corrected draft versions, and approved the final version.
